# Off-Label Medication: From a Simple Concept to Complex Practical Aspects

**DOI:** 10.3390/ijerph181910447

**Published:** 2021-10-04

**Authors:** Carmen-Maria Rusz, Bianca-Eugenia Ősz, George Jîtcă, Amalia Miklos, Mădălina-Georgiana Bătrînu, Silvia Imre

**Affiliations:** 1 Doctoral School of Medicine and Pharmacy, I.O.S.U.D., George Emil Palade University of Medicine, Pharmacy, Science, and Technology of Târgu Mureș (UMPhST), 540142 Târgu Mureș, Romania; carmenrusz20@gmail.com (C.-M.R.); batrinumadalina@yahoo.com (M.-G.B.); 2 Department of Pharmacology and Clinical Pharmacy, Faculty of Pharmacy, George Emil Palade University of Medicine, Pharmacy, Science, and Technology of Târgu Mureș (UMPhST), 540142 Târgu Mureș, Romania; george.jitca@umfst.ro; 3 Department of Biochemistry, Faculty of Pharmacy, George Emil Palade University of Medicine, Pharmacy, Science, and Technology of Târgu Mureș (UMPhST), 540142 Târgu Mureș, Romania; amalia.miklos@umfst.ro; 4 Department of Analytical Chemistry and Drug Analysis, Faculty of Pharmacy, George Emil Palade University of Medicine, Pharmacy, Science, and Technology of Târgu Mureș (UMPhST), 540142 Târgu Mureș, Romania; silvia.imre@umfst.ro

**Keywords:** off-label, practice, clinical, safety, regulation

## Abstract

Off-label use of drugs is widely known as unapproved use of approved drugs, and it can be perceived as a relatively simple concept. Even though it has been in existence for many years, prescribing and dispensing of drugs in an off-label regimen is still a current issue, triggered especially by unmet clinical needs. Several therapeutic areas require off-label approaches; therefore, this practice is challenging for prescribing physicians. Meanwhile, the regulatory agencies are making efforts in order to ensure a safe practice. The present paper defines the off-label concept, and it describes its regulation, together with several complex aspects associated with clinical practices regarding rare diseases, oncology, pediatrics, psychiatry therapeutic areas, and the safety issues that arise. A systematic research of the literature was performed, using terms, such as “off-label”, ”prevalence”, ”rare diseases”, ”oncology”, ”psychiatry”, ”pediatrics”, and ”drug repurposing”. There are several reasons for which off-label practice remains indispensable in the present; therefore, efforts are made worldwide, by the regulatory agencies and governmental bodies, to raise awareness and to ensure safe practice, while also encouraging further research.

## 1. Introduction

Off-label practice is a concept that has been in existence for many years. This concept has raised more and more awareness over the past years due to clinical advantages and liability perspectives, with the main focus on the patient’s safety. Although off-label practice precedes the conventional therapies and prescribing practices, there was neither awareness nor regulation regarding this matter. A turning point in the regulation of the medical and pharmaceutical field began around the tragedy due to the thalidomide use in the 1960s [1]. Regulation and control organizations started to impose more stringent conditions for a drug in order to grant marketing approval. The off-label practice was first recognized in the European Directive 2010/84/EU, which addresses the responsibility of marketing authorization holders (MAHs) to continuously monitor the drug product and provide all the available information, including the ones that are not in the scope of the marketing authorization. Moreover, this directive emphasizes the adverse events associated with this practice, encouraging the MAHs to redefine and to clarify the phrase ‘adverse event’ as to comprise the effects associated with the usage outside the specifications of Summary of Product Characteristics (SmPC), misuse/medication errors or drug abuse [2]. Attempts have been made either by European Committee (EC) or other governmental agencies in order to provide a comprehensive overview of the off-label picture from a legal and regulatory perspective.

A recent report from EC on off-label use indicates that some of the driving factors for off-label use are related to the marketing authorization process (long-development times and high costs, limited incentives for investigating new indications), post-marketing authorization events, pricing and reimbursement, and factors that concern the patients. In addition, specific cases have been indicated in which not only one, but a combination, of the aforementioned factors leads to the off-label practice [3].

Perhaps the most important aspect regarding the off-label practice, and one of the main advantages, is that it fulfills the unmet medical needs by the conventional therapeutic approaches, increasing the access to medication for special categories of patients. In agreement with the EC report on off-label use, the literature reveals that off-label practice is widespread in rare diseases [4,5,6,7,8,9,10,11], oncology [12,13,14,15,16], pediatrics [17,18,19,20,21,22,23], and psychiatry (especially in pediatric and elderly populations) [24,25,26,27,28,29,30,31,32,33,34,35,36]. It is difficult to establish a prevalence or a pattern of general off-label prescribing, since it embraces many forms, and it is divided into many categories. The most common categories of off-label use are: modification of dosage regimen, administration route, pharmaceutical form, different indication, different age group, and different categories of patients.

The awareness raised around the off-label concept is mostly captured through the position of the regulatory agencies. Worldwide, efforts are being made in order to either limit the off-label use, such as programs dedicated to the MAHs, encouraging them to conduct further research by offering incentives, or to ensure its safely practice: national regulations [3,37,38,39]. Given the above considerations, it is important to understand the implications and the challenges associated with the off-label use in those therapeutic areas that lack proper treatment or in which the conventional therapeutic approaches do not fulfill the patient’s needs. The safety concerns are an important part of the off-label process, as the lack of information on the benefit-risk ratio of the off-label use can lead to unpredictable outcomes of the treatment.

We conducted a systematic research in the PubMed, PLOS one, Cochrane library, and Google scholar databases. The search term was initially ”off-label use”. Based on prior knowledge on the subject, and considering the numerous articles in the literature containing keywords related to the off-label use, the search was extended to include the following: ”prevalence”, ”rare diseases”, ”oncology”, ”pediatrics”, ”psychiatry”, and ”drug repurposing”. The time-frame was set from 2005 until present. Articles in English, French, and Romanian language were taken into consideration. Reviews, randomized control trials, clinical trials, and case series were analyzed, and the inclusion criterion was the off-label designation of the drug product, regardless of the outcome of the treatment. The regulatory agencies websites of Food and Drug Administration (FDA), European Medicines Agency (EMA), and National Drug Agency and Medical Devices from Romania (ANMDM) were consulted.

## 2. Defining the Off-Label Concept

Off-label practice occurs when a drug is prescribed, dispensed, and administered on other terms than the ones specified in the SmPC by the MAH. Therefore, it embraces the uses of a medicinal drug product that are not foreseen in the SmPC, which is authorized/licensed for use in a particular country [40]. The off-label use should be clearly distinguished from the unlicensed/unauthorized use of a product; the latter refers to the use of a medicinal drug product in a country where no marketing authorization was granted by a relevant licensing authority, even if the respective drug product is licensed in other countries. Relevant regulatory agencies (e.g., national drug agencies, EMA, FDA) approve the MAHs to market the drug product in a country under specified conditions [40]. According to some definitions, unlicensed use would also comprise the modification of the pharmaceutical form of an approved drug product, even though, generally, this practice is embraced by the off-label concept [41].

With regard to the ways in which the off-label practice can be achieved, it is important to mention that sometimes it can occur in an unnoticed manner. For every drug product, the approved indications and conditions of use are foreseen in the SmPC; therefore, off-label could equal the slight modification of these approved terms (indications, route of administration, dosage, etc.).

The off-label prescribing occurs almost without exception in an intended manner. Some of the most important reasons are related to the unmet medical needs in a certain therapeutic area, the special groups of population that, for legal, ethical, and practical regards, have not been studied (children, pregnant and breastfeeding women, geriatric population), the lack of adequate pharmaceutical formulations, the absence of therapeutic alternatives, due to a patient’s individual physiological or pathological characteristics (co-morbidities, organ impairment), and for the cases where the conventional pharmacotherapy has failed [42].

## 3. Regulatory Framework

This practice is neither encouraged nor forbidden by the regulatory bodies. FDA states that, in prescribing and dispensing, according to good medical/pharmaceutical practice, health specialists must use legally approved medication; if a drug is prescribed outside the SmPC, it is their responsibility to be well informed about the product. This means that, before doctors decide in favor of off-label prescribing, they should perform a thorough investigation, taking into consideration the possible risks and side effects [43]. With these conditions respected, FDA shows support for off-label use. The American Medical Association (AMA) considers the off-label process a doctor’s right to decide for a patient’s benefit, also an innovation of the medical act. It is important to mention that off-label prescribing and dispensing does not represent clinical research nor experiments on humans of any kind [44].

In Europe, the main attitude is toward increasing the safety and decreasing the risk, even in off-label use situation. The purpose of EC is to ensure that every drug is marketed for its intended use, dosage, and route of administration. Off-label practice is neither forbidden nor authorized by the European pharmaceutical law, but it does not impede the physician to prescribe a medication in an off-label manner under his own responsibility. This attitude is toward offering the access to medication, as limiting the use of proven effective and safe medication would be unreasonable [41]. Unlike the United States’ general opinion on off-label process, in Europe, there are divided impressions on this matter between the individual member states. Several European countries have taken measures in order to control the off-label process and ensure its safe practice, i.e., Italy, Spain, Germany, the Netherlands, Sweden, Greece, Hungary, United Kingdom, and Lithuania [3]. These countries either issued specific legislation or created a regulatory frame in which this practice can be done with control. One interesting approach has been taken by France, where a Temporary recommendation for use (RTU) for a certain drug product is issued by the Agence Nationale de Sécurité du Médicament (ANSM) when the situation deems it necessary for the patient. Firstly, several factors are being taken into consideration before this RTU can be issued: the absence of available alternative treatment (same active substance, same dosage, and same pharmaceutical form), the scientific knowledge, the drug safety, the severity of disease, and the frequency of disease’s occurrence. Second, the MAH is requested by the ANSM to provide all of the available information on the respective indication. In this manner, the off-label practice is monitored through a protocol toward a safer use, while the knowledge regarding the drug product’s off-label use is improved. Amongst the countries that have not implemented any specific measures or regulation regarding the off-label use are: Austria, Belgium, Bulgaria, Denmark, Czech Republic, Finland, Portugal, Estonia, Malta, and Slovenia [3]. There are encouraging or discouraging arguments on this practice; yet, the central idea remains that off-label falls under the responsibility of the prescriber, and it is the patient’s right to request alternative on-label treatments if the off-label approach does not feel convenient [42].

In Romania, ANMDM has raised awareness regarding off-label use due to an unfortunate event of safety nature. Bevacizumab, an anti-vascular endothelial growth factor (VEGF), is a substance approved for treating various types of cancer, including breast, colorectal, pulmonary, or ovarian. For several years, though, it has successfully been used in ophthalmology in the treatment of age-related macular degeneration, as given in single dose, intravitreal administration. The risks associated with this practice would be several, including blindness, serious systemic adverse events, heart failure, and thrombotic events [45]. Thus, a case of blindness associated with off-label use of bevacizumab was reported. The prescribing doctor was accused of malpractice. In the matter, ANMDM issued a statement explaining the off-label use, potential benefit, responsibilities of parts, and potential risks. According to the Romanian institution, off-label is a legal practice, and full responsibility for it is taken by the health specialists involved in the process [39].

In an attempt to optimize the current off-label process and to emphasize safety, some authors suggested publishing off-label treatment guidelines. These guidelines would facilitate the individual documentation of a prescribing doctor; yet, it would not neglect the thoroughness of the process and the relevance of the studies that are important for the respective search [1]. The availability of so many studies in the literature regarding the manner of practice may encourage off-label prescribing, but it can also be a step ahead for further treatment options. This concept is entitled ”Evidence-based Medicine”, and it represents the best utilization of the current knowledge toward the benefit of the patient [42]. The EC also indicates that the aforementioned French system for submitting RTUs can be considered a good initiative.

## 4. Implications of the Off-Label Use in Certain Therapeutic Areas

As the literature shows extended off-label practice in the investigated therapeutic areas: rare diseases, oncology, pediatrics, and psychiatry, some examples of substances used in such manner are exemplified in Table 1. It can be observed that, in some cases, for one drug substance, off-label occurs concomitantly for more than one category (e.g., indication and dose).

### 4.1. Rare Diseases

Rare diseases are represented by such small populations that the costs for conducting clinical studies and development are immense. According to the definition of EC on rare diseases, these are ‘life-threatening or very serious conditions that affect no more than 5 in 10,000 people in the European Union’ [46]. FDA defines rare diseases as ‘a disease or condition affecting less than one in 200,000 people’ [47].

Given the circumstances, the treatment approach in rare diseases is mostly covered by off-label practice. There are examples in the literature in which certain drug substances administered in an off-label manner were successfully used in the treatment of rare diseases [4,48,49,50], although safety issues associated with the off-label uses have been reported, as well after treatment discontinuation [4,51].

According to Attwood et al., the orphan drug development is underrepresented in oncology research [52]. Specific types of cancer qualify as rare diseases. According to the Information Network on Rare Cancers, there are 198 types of cancer that are classified as rare diseases [53], and the therapeutic approach in this area is characterized by a low-level of treatment centralization and a widespread off-label use of medication [54,55]. A retrospective study conducted at the National Cancer Center Hospital in Japan examined the frequency of and indications for off-label use based on the number of requests, considering the applications submitted to an internal committee. The assessment of types of malignancies revealed that the majority of the applications requesting off-label use aimed to treat rare cancers, such as sarcoma, urachal cancer, bladder cancer (urologic cancer types), and thymic carcinoma [56]. The importance of exploiting the off-label use of drugs in rare cancers in order to obtain data is also emphasized by Casali et al. [54].

The number of rare diseases increases yearly due to advancement in diagnostic technologies, which triggers the development of molecular-targeted therapies or therapeutic drugs that target the human genome [46,57]. The analysis conducted by Attwood et al. [52] shows an increasing number of New Molecular Entities (NMEs) and orphan drug designations throughout the years. While scientific progress creates grounds for developing NMEs, another possible explanation of the increased interest in developing such drugs is related to the incentives provided by the regulatory agencies and the high economic revenues [58].

Thus, it is of paramount importance to obtain approved therapies in rare diseases, and this could be supported by the current off-label practice. Clinical trials are being conducted on the basis of positive results obtained following off-label treatment in rare diseases [11,59]. The literature offers an example in which the off-label use of a drug substance triggered its approval for the new indication: tocilizumab, approved for chronic inflammatory diseases was found to be very effective in Castleman’s Disease. As a result, it was approved in Japan for Castleman’s Disease treatment [48].

As part of improving the manner in which off-label is practiced, an initiative of the EC was taken in recently in order to encourage the MAHs to conduct further research: The Orphan Drug Regulation. Regulatory bodies are currently encouraging pharmaceutical companies to initiate studies in this segment in order to increase safety, by offering incentives of six months to two years of patent extension (six months of Supplementary Protection Certificate and two years of Patent Right Extension if the studies are performed for orphan medication, according to Orphan Drug Regulation in European Union) [1]. Besides market exclusivity, other incentives include protocol assistance, provision of scientific advice, access to the centralized authorization procedure, reduced fees for regulatory activities, some administrative and procedural assistance from the small- and medium-sized enterprises office of EMA, and opportunity to have access to grants from the EC and other sources [60]. In addition, the FDA, through the Office of Orphan Drug Development, provided such incentives as market exclusivity, research and development grants, tax credits, waiving of regulatory fees in order to support the early development, and a shorter time-frame to approval and marketing [61].

### 4.2. Oncologic Patients

Although rare cancer therapy constitutes a high proportion of the oncological off-label treatment, as discussed in the above paragraph, the off-label regimen is also frequent in case of patients suffering from common types of cancer. Several studies from the literature show that 50% of the cancer therapy occurred in an off-label regimen [62,63]. The multiple risk factors that lead to the development of malignant cells, the insidious onset and rapid changes of the pathogenesis mechanisms (genetic mutations), make cancer treatment a challenging practice. Chemotherapy remains the standard approach; still, it can become off-label, i.e., if the respective drug is used in another type of cancer than it is approved for. As it occurs in common practice, a monoclonal antibody/another agent can be augmented to the standard chemotherapy in an off-label regimen, but the main chemotherapy remains on-label [64,65]. Biological therapies with monoclonal antibodies show a promising pathway in suppressing the cancer response, e.g., rituximab, bevacizumab, lenvatinib [14,15,16], but many types of cancer are still unresponsive to monotherapy, requiring combination regimens. Off-label therapy in cancer is, therefore, indispensable and will always be used, to a certain extent, for several reasons: clinical care of multiple tumor types and certain patient’s characteristics are not always covered by the prescribing information in routine practice, there are situations in which the practitioners are willing to try medication with uncertain evidence outside clinical trials for patients with advanced cancer or metastatic stage of the disease, and unawareness of the prescriber of the existence of approved drugs for certain types of cancer [62]. Drogovoz et al. [66] also discuss the difficulties of conducting clinical trials in this area, the high standards for evaluating clinical trials of these drugs, and lack of proper incentives when the approval for new indications is intended. Another problem identified by the authors would be several approved treatment protocols for one type of tumor at the same time [66]. Moreover, there are matters regarding the costs of anticancer therapy, which are very high.

### 4.3. Pediatric Patients

The current focus is, nonetheless, on pediatric patients. Due to the lack of pediatric formulations, a lot of substances are prescribed to new-born children and adolescents in an off-label manner. In pediatrics, off-label treatment represents approximately one half of the total number of recommendations, according to some studies [17,67]. The European Commission estimates that pediatric medication covers over one hundred million children in Europe [1], which is a major concern of the off-label use.

As an overview, studies regarding off-label prevalence of prescribing took place in different hospitals in Europe. In a neonate unit in Spain, over a three-month period, 44.1% of the administered medication was off-label [67]. Another study from Brazil reported on a higher time interval (six months), a percentage of 95.6% prevalence of off-label prescribing in children [68]. Under the same circumstances, off-label practice in a Romanian hospital found the prevalence of prescribing to be 54%, especially in the dosage regimens [17]. These findings are consistent with literature reviews on off-label prevalence in pediatrics, which indicated ranges from 1.2 to 99.7%, with the highest rate in neonate intensive care unit settings [69].

As far as safety is concerned, a meta-analysis compared the efficacy and safety profile of these drugs in on-label and off-label administration in children. Interestingly, there were no significant differences in the frequency of adverse events between the compared on-label and off-label administrations [70]. In the pediatric oncology segment, the off-label prescribing has been analyzed throughout a retrospective study for a 10-year period. There was an increasing prevalence of off-label use during the study period, with an overall tolerability of use by the studied population, reflected in the small percent of treatment discontinuation. The authors offer, as a possible explanation, that the treatment often starts with a lower, more conservative dose, below the FDA-approved dosing [71]. Venekamp et al. [72] studied the efficacy and safety of antibiotics in middle ear infection (otitis media) in children. The results were unsatisfying, as at the end of the treatment, the pain symptoms were not relieved and the recurrence of the infection was the same as in the placebo group. An important finding was the elevated risk of developing gastro-intestinal reactions and allergic reactions [72].

Until the 1980s, clinical research in children was considered unethical and had been neglected. In the present, it is accepted that the safety must be increased in this area, and, just as do adults, children deserve equal chances of treatment. For this purpose, the Pediatric Regulation was founded in the year 2007. This body functions under the EC’s guidance, and it obliges the companies to conduct studies in children population through a Pediatric Investigation Plan (PIP), in order to increase the number of approved drugs for pediatric use [38]. The Regulation has very clear objectives: making possible and increasing high quality research studies for developing pediatric drugs and assuring the fact that most drugs in this segment are specifically designated for pediatric use, in proper dosage and pharmaceutical forms. Another objective would be facilitating the access to scientific information for research purposes regarding this matter [38].

Another important aspect of the off-label process in pediatrics is directed toward the parental informed consent, as the right to information and the informed consent are part of the patient’s rights [73,74]. Obtaining the informed consent is desirable from a liability perspective [75]. Many countries in Europe (France, Italy, the Netherlands, and Sweden, as well as the United Kingdom) implemented as a requirement, along with the off-label use, the informed consent of the patient [3]. According to the literature, a few patients were aware of the fact that their physician chose to prescribe an off-label product. This issue is particularly addressed in pediatrics, due to the unavailability of proper medication. Moreover, there is an increased awareness regarding safety associated with off-label use. The informed consent of the patient is not usually obtained by the prescriber prior to the initiation of the treatment. Balan et al. [76] conducted a meta-analysis regarding awareness of off-label prescribing in children, and the most important outcome was that few parents had knowledge of such treatment which their children were undergoing. Moreover, there were studies conducted amongst doctors and pharmacists. Pediatricians and neonatologists seemed to be the most familiar with this term when compared to general practitioners, but situations were identified in which doctors were not aware of the fact that they were prescribing off-label drugs [76,77,78,79]. The majority of the pharmacists were familiar with this practice, but this seemed to be a matter of professional experience [76]. An issue regarding the informed consent would be that patients lack medical education, and they sometimes do not completely understand why such an approach is needed. A study conducted in Germany revealed that, in the situation of being informed, 9% of the parents would refuse the treatment, and 51% of the parents would accept it only when they knew it was the last alternative [80].

### 4.4. Psychiatric Patients

The off-label use in psychiatry is well-documented, showing an increasing tendency over time [81], due to the ability of psychotropic drugs to treat multiple symptoms associated with these disorders. This approach targets mostly the special categories of patients, i.e., pediatric [81,82] and geriatric populations [83], which counts as off-label by a different age group, but it is also frequent in the category of off-label by indication [33,34,35,36]. The off-label use of psychotropic drugs is controversial, mainly due to its high prevalence in children, adolescents, and the elderly. In these populations, safety issues arise. Other important reasons that generate controversy are indicated by studies in the literature: the difficulty of measuring of the off-label phenomenon which can be over- or underestimated [84,85], the unjustified off-label prescribing with no evidence of superiority when compared with on-label medication (for example, antidepressants prescribed for sleeping show no evidence of superior efficacy compared with benzodiazepines [86]), or unjustified off-label prescribing by physicians of other medical specialties than psychiatry [87].

However, there are examples in which substances have been successfully used in off-label treatment. The most important safety regard in this category of patients is supported by the off-label use of trazodone for treating insomnia symptoms in a large group of patients, for a long period of time, which was well-tolerated [27,28,29]. Studies concerning psychotropic drugs indicate the fact that some of these are safe in off-label use in children, with a manageable profile of adverse events [82]. A meta-analysis conducted by Ray et al. [88] identified an increased risk of sudden death associated with antipsychotic medication in children and adolescents, emphasizing the careful prescribing and follow-up of the psychiatric treatment in this population [88].

## 5. Safety Initiatives

As we can infer from the problems discussed above, off-label use can sometimes be preferred for several reasons, or it can be the only therapeutic alternative for some patients. Therefore, in these situations, close monitoring of the side effects associated with off-label use is a prerogative. Studies in the literature support the importance to assess the safety of off-label prescribing in populations where randomized clinical trials are unlikely to be performed [89]. Monitoring the safety of off-label use of medicines can be done through several methods, which have been described by Dal Pan [90]: spontaneous reports, observational pharmacoepidemiologic studies, registries, and clinical trials. Any of these approaches could contribute to collecting a significant body of scientific data, in order to enhance a safe off-label practice. The side effects of drugs are usually reported in pharmacovigilance databases, and this also applies to the ones associated with off-label use. EudraVigilance, which is the pharmacovigilance system of EMA, identifies 820 serious adverse events, 130 being fatal, between 2001 and 2004 [91]. Pharmacovigilance databases were also nationally implemented and consulted with regard to the off-label use; databases from France and Italy contributed to the assessment of adverse events/side effects of drugs associated with off-label use on different populations [92,93]. However, there are concerns that there is an underreporting phenomenon occurring through national spontaneous reporting systems. [94]

Eguale et al. [95] emphasize the safety aspect of the off-label use in a study that compared the frequency of side effects correlated with off-label, respectively, on-label use. The information was retrieved from a database that contained all the electronic prescriptions (151,305 prescriptions) from Canada in a 4-year period. A percentage of 11.8% of the total prescriptions constituted an off-label indication. The adverse events correlated with these off-label indications were 40% more frequent than on-label recommendation cases. Moreover, it was stated that, in 80.9% of the cases, these recommendations were not made based on strong scientific evidence [95].

## 6. Closing Remarks and Future Directions

Taking all these facts into account, off-label use cannot be entirely suppressed; therefore, the regulatory agencies intend to shape this concept into becoming as safe as possible. The efforts reflected in pharmacovigilance practices are important, especially through the reporting, by the prescribing physician, of any experience, either successful or undesired. In this manner, a significant body of scientific data could be collected which would make off-label treatment predictable and, eventually, successful.

Off-label practice constitutes a starting point for future research and directions into marketing certain drug products for new indications, shifting to drug repurposing. There have been cases in which off-label use led to drug repurposing [48]; however, in most of the cases, the drug repurposing is triggered by serendipitous discoveries (e.g., thalidomide, sildenafil). Sildenafil has been originally approved for pulmonary hypertension and then repurposed for erectile dysfunction [96]. Contradictorily, off-label practice could also lead to off-label use. In the European regulatory framework, drug repurposing can be translated into clinical practice in two ways: approving the new indication and granting market authorization or allowing the new indication to be off-label, based on national regulations [62,97]. All these considered, a repurposed drug product will not necessarily be exempted from off-label use, i.e., a pharmaceutical company approves the new indication, yet the availability of other generic drugs can still lead to off-label use of the generic drugs, if the approved drug is not specifically requested as prescriber’s means to an end [97]. Furthermore, if a certain company does not invest in developing the new therapeutic indication, the off-label prescribing of their product may be beneficial, as it expands the addressability to new categories of patients, without having to apply for a new marketing authorization [97].

To conclude, the off-label concept, which embraces multiple aspects, mainly of clinical, regulatory, and safety nature, requires constant awareness and responsibility of its practice and, more importantly, the collaboration of regulatory bodies, MAHs, healthcare specialists, and, not lastly, the patients.

## Figures and Tables

**Table 1:** Example of substances that are used in an off-label manner.

Active Drug	Therapeutic Area	Manner of Off-Label Use	Approved Use According to SmPC	Off-Label Use	Reference
Denosumab	Rare diseases	Indication	Osteoporosis in postmenopausal women and in men at increased risk of fractures Treatment of bone loss associated with hormone ablation in men with prostate cancer at increased risk of fractures Treatment of bone loss associated with long-term systemic glucocorticoid therapy in adult patients at increased risk of fracture	Juvenile Paget Disease Fibrous dysplasia Hypophosphatasia Pregnancy and lactation osteoporosis Lagerhans cell hystiocytosis	[4,5] [4,6] [4,7] [4,8] [4,9]
		Category of patients	Adults	Children	[4,10]
Ruxolitinib		Indication	Myelofibrosis Polycythemia vera	Vitiligo	[11]
Metformin	Oncology	Indication	Adult type II diabetes, especially in overweight patients	Epidermal Growth Factor Receptor-Mutated Lung Adenocarcinoma	[12]
Simvastatin Lovastatin Atorvastatin Fluvastatin Pravastatin Rosuvastatin		Indication	Dyslipidemia Secondary prevention of coronary heart disease	Hepatocellular carcinoma	[13]
Adrenaline	Pediatrics	Route of administration	Intravenous/intramuscular/subcutaneous/intracardiac injection	Nebulization	[17]
Cloramphenicol and betahametasone		Route of administration	Intraocular administration	Intranasal administration	[17]
Spironolactone Captopril Enalapril Propranolol Furosemide		Pharmaceutical form	Oral tablets	Powder obtained from grinding the tablets	[18,19]
Sirolimus Everolimus		Indication	Prevention of acute rejection in kidney transplantation in adults	Immunologic disorders Proliferative disorders or vascular abnormalities Congenital hyperinsulinism	[20]
Carvedilol Metoprolol		Indication	Congestive heart failure in adults	Congestive heart failure in children	[21,22,23]
Mirtazapine	Psychiatry	Indication	Major depressive episodes	Avoidant and restrictive food intake disorder	[24]
		Category of patients	Adults	Adolescents	
Anastrozole Letrozole		Indication	Breast cancer in women	Height increment in boys	[25,26]
Trazodone		Indication	Depression associated with or without anxiety	Insomnia	[27,28,29,30]
Trazodone		Dosage	75–150 mg/day	50–100 mg/day	
Quetiapine		Indication	Schizophrenia, bipolar disorders, major depressive disorder	Insomnia	[31]
Prazosin		Indication	Treatment of arterial hypertension	Post-traumatic stress disorders	[32]
Ketamine		Indication	Anesthesia	Depression	[33,34]
Methylphenidate		Category of patients	Children	Adults	[35,36]
